# Factors Influencing Telemedicine Adoption Among Healthcare Professionals in Geriatric Medical Centers: A Technology Acceptance Model Approach

**DOI:** 10.3390/bs15101367

**Published:** 2025-10-07

**Authors:** Tammy Porat-Packer, Gizell Green, Cochava Sharon, Riki Tesler

**Affiliations:** 1 Shoham Medical Center, Pardes Hanna-Karkur 3701001, Israel; 2 Department of Nursing, The Max Stern Academic College of Emek Yezreel, Emek Yezreel 1930600, Israel; 3 Department of Nursing, Sha’arei Mishpat College, Margo’a St 5, Hod Hasharon 4510201, Israel; 4 Department of Nursing, Faculty of Health Science, Ariel University, Ariel 4070000, Israel

**Keywords:** telemedicine, technology acceptance, healthcare professionals, self-efficacy, social influence, technology anxiety, organizational behavior, structural equation modeling

## Abstract

Background: Telemedicine has gained significance, especially during the COVID-19 pandemic, offering remote healthcare solutions. However, its adoption in geriatric medical centers (GMCs) remains limited. Understanding the factors influencing telemedicine acceptance among care teams in geriatric medical centers is crucial for successful implementation. Aim: This study examines behavioral factors influencing telemedicine adoption among care teams in Israeli geriatric medical centers through the lens of the Technology Acceptance Model. Methods: A cross-sectional study was conducted with 406 healthcare professionals from four geriatric medical centers in Israel. Participants completed a self-administered questionnaire measuring self-efficacy, subjective norms, anxiety, resistance to change, perceived usefulness, perceived ease of use, and intention to use telemedicine. Structural equation modeling was used to analyze the data. Results: Perceived ease of use mediated the associations between self-efficacy and perceived usefulness and between subjective norms and perceived usefulness, demonstrating how confidence shapes technology acceptance. Perceived usefulness mediated the association between perceived ease of use and intention to use. Perceived ease of use did not mediate the relationship between anxiety or resistance to technological changes and perceived usefulness. Conclusions: The study highlights the importance of perceived ease of use and usefulness in promoting telemedicine adoption among geriatric medical center care teams, emphasizing the need for targeted interventions to enhance these perceptions.

## 1. Introduction

Israel’s healthcare system serves 9.5 million people with universal coverage through four competing Health Maintenance Organizations. Despite having only 3.3 physicians and 5.1 nurses per 1000 population (below OECD averages) Israel maintains a life expectancy of 82.6 years (Levi & Davidovitch, 2022). The aging population (11.2% over 65) faces critical challenges: long-term care beds decreased from 3.97 to 2.9 per 1000 elderly individual between 2010 and 2021, with significant geographic disparities physician density in Tel Aviv (5.8/1000) more than doubles that in peripheral regions (Rotenberg et al., 2022). Telemedicine offers a promising solution to bridge these gaps in geriatric care accessibility.

### 1.1. Telemedicine: Definition and Benefits

Telemedicine enables remote healthcare delivery through digital technologies, allowing providers to evaluate, diagnose, and treat patients without in-person visits (Kuan et al., 2022). Telemedicine provides high-quality care while improving access for underserved populations, particularly in peripheral and rural settings (Boehm et al., 2020; Driggin et al., 2020; Price & Simpson, 2022; Verfürth, 2020). This capacity is especially relevant for geriatric populations facing mobility challenges (Coppini et al., 2025). Research demonstrates telemedicine benefits for elderly patients including improved quality of life, reduced hospitalizations, and economic savings (Graham & Jones, 2020; Haimi & Gesser-Edelsburg, 2022; Zulfiqar et al., 2018). Global adoption has increased exponentially (Bashshur et al., 2020), with widespread implementation in the United States (Centers for Disease Control and Prevention, 2020), United Kingdom (World Health Organization, 2021), and Israel where COVID-19 guidelines support telemedicine use (Grossman et al., 2020). Despite documented benefits, behavioral barriers impede adoption in geriatric medical centers where established care routines strongly influence practice patterns.

### 1.2. Challenges in Telemedicine Adoption

The behavioral challenges of telemedicine adoption are particularly stated in geriatric care settings, where healthcare professionals have developed specific interaction patterns suited to elderly patients’ complex needs. What distinguishes geriatric care contexts from general healthcare settings is the multidimensional complexity of patient needs, including cognitive impairments, multiple comorbidities, sensory deficits, and high dependency levels that require, personalized care approaches (Graham & Jones, 2020; Layfield et al., 2020). Unlike younger populations, geriatric patients often require tactile assessment, non-verbal communication, and environmental evaluation that are challenging to replicate through digital mediums. This creates a unique tension between the efficiency promised by telemedicine and the holistic, high-touch care traditionally valued in geriatric practice. The professionals caregivers must manage not only their own behavioral adaptation but also support patients who may struggle with technology (Layfield et al., 2020). The use of telemedicine largely depends on individuals’ digital health literacy levels (Coleman, 2020), creating a dual behavioral challenge where professionals must simultaneously manage their own technology-related behaviors and facilitate patient engagement. Despite the advantages and development of telemedicine, in the geriatric medical centers in Israel, there is currently limited use of it, which mainly, if any, includes consultations between caregivers and patients only during the COVID-19 pandemic, suggesting significant behavioral barriers beyond mere technological availability.

Studies in acute care settings show that telemedicine adoption primarily depends on efficiency gains and technical factors (Jacob et al., 2020) (Harst et al., 2019). However, in geriatric settings, relational factors may override technical considerations. While emergency and surgical departments successfully adopt telemedicine based on perceived usefulness alone (Almathami et al., 2020), we assumed that in GMCs, preserving therapeutic relationships may disrupt traditional Technology Acceptance Model (TAM) pathways, explaining differently the pathway of perceived ease of use (PEOU) and perceived usefulness (PU) typically found in other healthcare settings.

### 1.3. Theoretical Framework: Technology Acceptance Model (TAM)

Understanding technology adoption through a behavioral lens requires theoretical frameworks that capture the psychological and social factors influencing professional behavior change. Users’ willingness and desire to use technology are crucial factors in the successful adoption and utilization of technology (Aggelidis & Chatzoglou, 2008). The Technology Acceptance Model (TAM), developed by Davis (1989), has been extensively validated as one of the most reliable models of technology acceptance (Carter & Bélanger, 2005). We chose TAM over more recent models like The Unified Theory of Acceptance and Use of Technology 2 (UTAUT2) (Venkatesh et al., 2012) because TAM’s focus on the mediating role of PEOU directly addresses our research questions about behavioral barriers in geriatric care. Additionally, UTAUT2’s consumer-oriented constructs (e.g., hedonic motivation) are less relevant in GMCs. Originating in sociology and psychology, TAM provides a behavioral framework for understanding how individuals form intentions to use new technologies based on their perceptions and beliefs (Jackson et al., 2013).

The TAM model consists of two main constructs: PEOU and PU. PEOU refers to the degree to which an individual believes that using technology will be effortless, while PUs refers to the degree to which an individual believes that using technology will improve their job performance (Davis, 1989). From a behavioral perspective, these perceptions mediate the relationship between external psychological factors and actual technology use behaviors (Guo et al., 2013; Saadé & Kira, 2009; Tsai et al., 2020). The intention to use represents the degree to which individuals intend to incorporate technology into their work behaviors (Davis, 1989). In geriatric settings, these constructs may operate differently due to the complex interplay between technological efficiency and the preservation of therapeutic relationships that are particularly crucial for elderly patients’ wellbeing.

### 1.4. Behavioral Factors Influencing Telemedicine Adoption

This study examines behavioral factors influencing telemedicine adoption among GMC healthcare teams. Promoting factors are self-efficacy, belief in one’s ability to perform tasks (Bandura, 1981; Compeau & Higgins, 2017) and subjective norms, perceived social pressure (Davis, 1989; Lazarus et al., 2021). Inhibiting factors include technology anxiety, fear toward technology (Maurer & Simonson, 1984), which negatively impacts PEOU and PU (Guo et al., 2013; Igbaria et al., 1996; Rabaa’i, 2016; Tsai et al., 2020) and resistance to change, comprising routine seeking, emotional reaction, short-term focus, and cognitive rigidity(Oreg et al., 2008) which correlates negatively with PEOU (Guo et al., 2013; Tsai et al., 2020). These relationships remain unexplored in GMC contexts.

Furthermore, PEOU may affect PU and behavioral intention to use, which in turn may determine whether a user will use the technology (Chavoshi & Hamidi, 2019; Davis, 1989; Venkatesh et al., 2003). This behavioral chain reflects individuals’ tendency to adopt systems that provide benefits without excessive effort, a fundamental principle of behavioral economics applied to professional context (Bhattacherjee & Hikmet, 2007).

The unique behavioral context of geriatric medical centers requires special consideration. Caregiver populations in GMCs work across departments for long-term geriatric care, active geriatric care in complex clinical settings, prolonged ventilation, supportive care (hospice), sub-acute geriatrics, and rehabilitative geriatrics. These diverse care contexts create varied behavioral norms and practice patterns that differ from those in general hospitals or community settings. The heterogeneity of care needs within GMCs—ranging from cognitively intact patients requiring rehabilitation to those with advanced dementia requiring palliative care—demands flexible technology adoption strategies that traditional TAM applications may not fully address. Furthermore, the interdisciplinary nature of geriatric care teams, including physicians, nurses, social workers, and therapists, creates complex social dynamics that influence technology acceptance through varied professional cultures and hierarchies (Almathami et al., 2020). Understanding the behavioral factors influencing telemedicine acceptance in this specialized context is essential for developing targeted interventions.

### 1.5. Study Aims and Hypotheses

Despite the extensive application of TAM in healthcare settings, its application in geriatric medical centers remains unexplored, particularly regarding the unique behavioral dynamics that characterize elderly care. Accordingly, the purpose of the study is to examine the promoting and inhibiting factors (self-efficacy for using telemedicine, subjective norms towards telemedicine, anxiety about using telemedicine, and resistance to technological changes at work) and PU and PEOU of telemedicine, as mediating variables for intentions to use telemedicine among caregivers from government geriatric medical centers. Accordingly, the research hypotheses are:

Based on research demonstrating that self-efficacy enhances PEOU (Guo et al., 2013) in mobile health contexts and that PEOU influences PU (Venkatesh et al., 2003) across diverse technological implementations we hypothesize that:

1. Perceived ease of use of telemedicine will mediate the relationship between self-efficacy for using telemedicine and the PU of telemedicine in the geriatric care context.
  Following studies showing that subjective norms positively affect technology acceptance through PEOU (Lazarus et al., 2021; Schepers & Wetzels, 2007) in healthcare professional communities we hypothesize that:
2. Perceived ease of use of telemedicine will mediate the relationship between subjective norms towards telemedicine and the PU of telemedicine among interdisciplinary geriatric care teams.
  Given findings that technology anxiety negatively impacts PEOU (Guo et al., 2013; Tsai et al., 2020), we hypothesize
3. Perceived ease of use of telemedicine will mediate the relationship between anxiety about using telemedicine and PU in the specialized geriatric care environment.
  Based on evidence that resistance to change correlates negatively with PEOU (Guo et al., 2013; Liao et al., 2016), we hypothesize that:
4. Perceived ease of use of telemedicine will mediate the relationship between resistance to technological changes at work and the PU of telemedicine in government geriatric medical centers.
  Consistent with TAM’s core proposition that PU mediates between PEOU and intention to use (Chavoshi & Hamidi, 2019; Davis, 1989) and extending this to the unique demands of geriatric care where efficiency must balance with relationship-centered care we hypothesize that:
5. PU of telemedicine will mediate the relationship between PEOU of telemedicine and intention to use telemedicine in government geriatric medical centers.

## 2. Methods

### 2.1. Participants

The study included 406 healthcare professionals from four government geriatric medical centers in Israel: Shoham, Dorot, Fliman, and Shmuel Harofeh. Participants represented diverse professional roles including physicians, nurses, physiotherapists, occupational therapists, social workers, speech therapists, and clinical dietitians, enabling examination of behavioral patterns across different professional cultures.

Sample size was calculated based on the total population of 1086 healthcare professionals in the four GMCs (146 physicians, 790 nurses, 248 allied health professionals). Using a 95% confidence level, 50% population proportion, and 4.4% margin of error, the minimum required sample was 341 participants; however, we recruited 406 participants to ensure adequate power for the complex SEM analysis.

Inclusion criteria: Healthcare professionals employed ≥6 months (ensuring familiarity with organizational norms), Hebrew proficiency, and informed consent. Convenience sampling with deliberate efforts to achieve representative coverage across professional groups, departments, and work shifts (Bornstein et al., 2013; Sedgwick, 2013).

### 2.2. Procedure

Data collection (March–June 2024) was designed to minimize social-desirability bias. Trained health sciences students conducted surveys according to pre-arranged departmental schedules. Anonymous self-report questionnaires were immediately sealed in envelopes to ensure confidentiality and encourage honest reporting of technology-related anxieties.

### 2.3. Instrument

The study employed a self-administered questionnaire composed of eight parts:

1. Self-efficacy for using telemedicine: This variable was measured using a 4-item scale adapted from Venkatesh et al. (2003). Example item: “I could complete a job or task using telemedicine if there was no one around to tell me what to do as I go.” Participants rated their self-efficacy on a scale from 1 to 10, with 1 indicating “not at all confident,” 5 indicating “moderately confident,” and 10 indicating “completely confident” (Venkatesh et al., 2003). The internal consistency reliability found was 0.87.
2. Subjective norms towards telemedicine: This variable was measured using a 2-item scale from Venkatesh et al. (2003). Example item: “People who influence my behavior think that I should use telemedicine in my work.” A 7-point Likert scale was used, ranging from 1 “strongly disagree” to 7 “strongly agree” (Venkatesh et al., 2003). The internal consistency reliability found was above 0.70.
3. Anxiety about using telemedicine: This variable was measured using a 4-item scale from Venkatesh et al. (2003). Example item: “I feel apprehensive about using telemedicine in my work.” A 6-point Likert scale was used, ranging from 1 “strongly disagree” to 6 “strongly agree” (Venkatesh et al., 2003). The internal consistency reliability was 0.82.
4. Resistance to technological changes at work: This variable was measured using a 17-item scale developed by Oreg et al. (2008), consisting of four categories: routine seeking, emotional reaction to change, short-term focus, and cognitive rigidity. Example item: “When my work procedures change, it seems like a real hassle to me.” A 6-point Likert scale was used, ranging from 1 “strongly disagree” to 6 “strongly agree.”. The internal consistency reliability was 0.85.
5. PU of telemedicine: This variable was measured using a 6-item scale adapted from Davis (1989). Example item: “Using telemedicine would be useful in my job.” A 7-point Likert scale was used, ranging from 1 “strongly disagree” to 7 “strongly agree” (Davis, 1989). The internal consistency reliability found was above 0.70.
6. Perceived ease of use of telemedicine: This variable was measured using a 6-item scale adapted from Davis (1989). Example item: “I would find it easy to use telemedicine in my work.” A 7-point Likert scale was used, ranging from 1 “strongly disagree” to 7 “strongly agree” (Davis, 1989). The internal consistency reliability was above 0.70.
7. Intention to use telemedicine: This variable was measured using a 3-item scale from Davis (1989). Example item: “I intend to use telemedicine technology in my work in the near future” (Davis, 1989). A 7-point Likert scale was used, ranging from 1 “strongly disagree” to 7 “strongly agree.” The internal consistency reliability was 0.89.
8. Demographic and Background Data: Demographic and professional background data were collected to contextualize behavioral patterns. Personal demographics included gender, age, marital status, parental status (including number of children), religion, and level of religiosity, capturing personal and cultural factors that may influence technology adoption behaviors. Professional characteristics encompassed job role (physician, nurse, physiotherapist, occupational therapist, social worker, speech therapist, clinical dietitian), years of professional experience, and work schedule patterns (morning only versus rotating shifts), etc. The back-translation process was conducted by professional translators, including one native English speaker who verified the accuracy and linguistic appropriateness of the final English version.

Before data collection, a pilot study was conducted among a small group matching the characteristics of the study population to test the validity and reliability of the questionnaires among caregivers in GMCs. The pilot study demonstrated good internal consistency reliability for all questionnaires, with Cronbach’s alpha values ranging from 0.70 to 0.89.

### 2.4. Data Analysis

Data analysis employed contemporary structural equation modeling approaches suited to testing complex behavioral pathways. Analyses were conducted using SPSS version 27 and R software version 4.2.1 (‘lavaan’ package). Analyses included descriptive statistics examining variable distributions, normality tests (Shapiro–Wilk, Kolmogorov–Smirnov, and Anderson–Darling), confirmatory factor analysis (CFA) validating the measurement model, structural equation modeling testing hypothesized pathways, Harman’s single-factor test assessing common method bias, Fornell–Larcker criterion for discriminant validity, McDonald’s omega for composite reliability and Average Variance Extracted (AVE) for convergent validity bootstrapping procedures (5000 samples) for robust mediation testing, and multi-group analyses examining gender-based model invariance across professional groups. Model fit was assessed using multiple indices appropriate for research: *χ*^2^/df < 3.0, CFI > 0.90, TLI > 0.90, RMSEA < 0.08, SRMR < 0.08, ensuring comprehensive evaluation of the proposed behavioral model.

### 2.5. Ethical Considerations

The study received IRB approval from School of Health Sciences Ariel University. All participants provided informed consent and were assured confidentiality. Participation was voluntary, with withdrawal rights preserved. Data were stored securely with restricted access; results published in aggregate form only.

## 3. Results

The average age of the study participants was 40.39 (SD = 11.49) years, and the average number of years of professional experience (tenure) was 12.58 (SD = 11.30) years. For other background characteristics, see Table 1.

Table 1 shows that the majority of participants were women and the rest were men, most of whom were married or in a relationship. Most participants were secular or traditional, with a minority being religious or very religious. Regarding religion, most participants were Jewish, minors were Druze, and 3 participants defined their religion as “other”. In addition, most study participants were parents. Concerning a job description, about half of the samples described their role as a nurse, while the rest were physicians, physiotherapists, occupational therapists, social workers, clinical dietitians, and speech therapists, respectively. Finally, about half of the participants work in shifts, and about a third of the participants work morning hours.

Initial statistical tests (Shapiro–Wilk, Kolmogorov–Smirnov, and Anderson–Darling tests) revealed that the study variables were not normally distributed. For the structural equation modeling, a robust estimator was used to account for the non-normal data distribution.

Discriminant validity was assessed using the Fornell–Larcker criterion (Fornell & Larcker, 1981). See Table 2.

Table 2 shows that discriminant validity was confirmed using the Fornell–Larcker criterion, with the square root of AVE for each construct (shown in bold on the diagonal) exceeding all correlation values with other constructs.

To test the proposed mediation model and examine the relationships among the promoting factors, inhibiting factors, perceived usefulness, perceived ease of use, and intention to use telemedicine, confirmatory factor analysis (CFA) and structural equation modeling (SEM) were conducted using R software (‘lavaan’ package).

The CFA model generated good fit indices: *χ*^2^(715) = 1763.77, *p* < 0.001; *χ*^2^/df = 2.47; TLI = 0.918; CFI = 0.925; RMSEA [90% C.I.] = 0.061 [0.058, 0.065]; SRMR = 0.068. Since all data were collected through self-administered at a single time point, two statistical tests were conducted to ensure that the collected data did not exhibit characteristics of a single general factor. To assess the potential for common method bias, we conducted Harman’s single-factor test (Podsakoff et al., 2003). All items from the study constructs were entered into an unrotated principal components factor analysis. The results showed that the first factor accounted for 36.70% of the variance, which is below the 50% threshold that would indicate substantial common method bias.

Furthermore, reliability analysis revealed satisfactory to excellent internal consistency across all factors. Composite reliability coefficients, calculated using McDonald’s omega, were consistent with these findings, ranging from 0.75 to 0.97. Convergent validity was assessed using Average Variance Extracted (AVE) values. All factors demonstrated adequate convergent validity with AVE values above the recommended 0.50 threshold.

**Figure 1:**
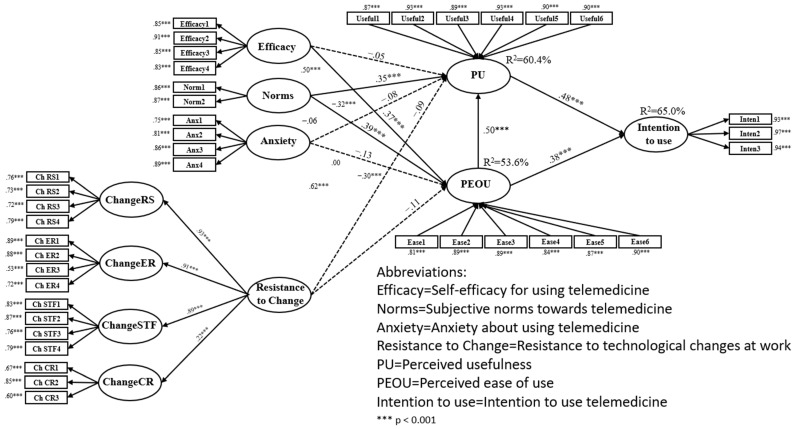
Research model.

Also, the SEM model generated good fit indices: *χ*2(719) = 1783.77, *p* < 0.001; *χ*2/df = 2.48; TLI = 0.917; CFI = 0.924; RMSEA [90% C.I.] = 0.061 [0.057, 0.064]; SRMR = 0.068. Figure 1 illustrates the structural model, indicating estimated path coefficients and the percentage of explained variance. For the model data, see Figure 1. 

To test whether PEOU mediated the associations between self-efficacy for using telemedicine, subjective norms towards telemedicine, anxiety about using telemedicine, and resistance to technological changes at work, and perceived usefulness, and whether PU mediated the associations between PEOU and intention to use telemedicine, bootstrapping procedure (*n* = 5000) and 95% confidence intervals (CIs) were calculated.

Table 3 shows, perceived ease of use mediated the associations between self-efficacy for using telemedicine and PU(B = 0.17, SE = 0.04, CI 95% [0.09, 0.25]), and between subjective norms towards telemedicine and PU(B = 0.21, SE = 0.03, CI 95% [0.14, 0.28]), and PU mediated the associations between perceived ease of use and intentions (B = 0.29, SE = 0.05, CI 95% [0.19, 0.39]).

Figure 1 shows that intention to use telemedicine was predicted positively by PU and PEOU (R^2^ = 65.0%). PU was predicted positively by PEOU and subjective norms towards telemedicine (R^2^ = 60.4%). Lastly, PEOU was predicted positively by self-efficacy for using telemedicine and subjective norms towards telemedicine (R^2^ = 53.6%). Thus, the higher the self-efficacy in using telemedicine and subjective norms towards telemedicine, the higher the PEOU; the higher the subjective norms towards telemedicine and PEOU, the higher the PU; and the higher the PEOU and the PU, the higher the intention to use telemedicine.

### Multigroup Analysis Findings

The multigroup analysis indicated no significant differences in the structural path patterns between males and females. An examination of the model’s paths showed that none differed significantly in strength across groups (all *p*-values > 0.05). While some numerical variations were observed—for example, the path from Self Efficiency to Ease of use was stronger among females (β = 0.493) than males (β = 0.304), and the path from Anxiety to Ease of use was significant only for males (β = −0.187, *p* = 0.025) but not for females (β = −0.008, *p* = 0.936)—these differences did not reach statistical significance in the Chi-square difference tests. Likewise, the indirect (mediation) effects showed no significant group differences. Overall, these results suggest that the theoretical model functions similarly for males and females, and that the influence of the studied variables on technology usage intention is not meaningfully moderated by gender.

## 4. Discussion

The present study aimed to examine behavioral factors influencing telemedicine adoption among healthcare teams in Israeli geriatric medical centers through the lens of promoting factors (self-efficacy, subjective norms) and inhibiting factors (anxiety, resistance to change), with PU and PEOU as mediating variables. The diverse findings shed light on the complex behavioral mechanisms that may promote or inhibit telemedicine use intentions among healthcare teams in GMCs, providing a comprehensive picture of this important issue from a behavioral science perspective.

Consistent with the literature, the study found that higher self-efficacy for using telemedicine and subjective norms towards telemedicine were associated with higher PU and PEOU of telemedicine (Almathami et al., 2020; Bandura, 1981; Harst et al., 2019; Saadé & Kira, 2009; Schepers & Wetzels, 2007; Strong et al., 2006). These findings confirm that behavioral confidence and social influence operate as powerful facilitators of technology acceptance, shaping both cognitive evaluations and behavioral intentions. According to Bandura’s Social Cognitive Theory, self-efficacy influences behavior through four processes: cognitive, motivational, affective, and selection processes, which explains why confident professionals not only perceive technology as easier but also more useful in clinical practice (Bandura, 2001). The strength of these relationships underscores their practical significance for implementation strategies.

Conversely, higher levels of anxiety about using telemedicine and resistance to technological changes at work were associated with lower PU and PEOU, aligning with previous research (Guo et al., 2013; Igbaria et al., 1996; Rabaa’i, 2016; Tsai et al., 2020). These negative perceptions and fears often create the impression that telemedicine is threatening and unhelpful, leading to resistance to its adoption (Xue et al., 2015; Zander, 1950) From a behavioral perspective, these emotional responses represent protective mechanisms that maintain established professional practices and defend against perceived threats to competence.

The study also found that PEOU mediated the relationships between self-efficacy and PU, and between subjective norms and PU, as hypothesized and supported by other research findings (Harst et al., 2019; Shiferaw & Mehari, 2019). This mediation pattern reveals a critical pathway: positive psychological states must first reduce perceived complexity before professionals can recognize technology’s value. Venkatesh and Davis (2000) describe this as “internalization,” where social influences shape usefulness perceptions only after being processed through personal ease-of-use assessments (Venkatesh & Davis, 2000). PU was positively associated with intentions to use telemedicine and mediated the relationship between PEOU and intentions to use, providing validation and support for the TAM (Davis, 1989; Jacob et al., 2020; Killikelly et al., 2017). These findings demonstrate the sequential nature of behavioral change, where perceptual shifts precede intentional modifications in professional practice.

Contrary to expectations, the study found that PEOU did not mediate the relationship between anxiety or resistance to technological changes and PU. This unexpected finding represents one of our study’s most significant contributions, challenging the assumption that all behavioral factors operate through the same psychological pathways. This finding aligns with another study’s critique of TAM, suggesting that emotional factors may bypass cognitive appraisal processes in high-stakes healthcare contexts. This unexpected finding suggests that negative behavioral states operate through fundamentally different psychological mechanisms than positive facilitators (Bagozzi, 2007). Also, the multidimensional nature of resistance to change, comprising routine seeking, emotional reaction, short-term focus, and cognitive rigidity (Oreg et al., 2008), may contribute to this complexity, as these dimensions could function differently in geriatric contexts.

Three interconnected explanations emerge from our analysis. First, in GMCs, anxiety about telemedicine may stem from deeper concerns about compromising the quality of care for vulnerable elderly patients rather than simply technical difficulties. Healthcare professionals in geriatric settings prioritize tactile assessment, non-verbal cues, and holistic evaluation elements difficult to replicate digitally (Graham & Jones, 2020; Layfield et al., 2020). This reflects what other researchers term “sociotechnical complexity,” where technology adoption in healthcare involves navigating competing values between efficiency and traditional care quality (Greenhalgh et al., 2017). Thus, even if the technology becomes easier to use, these fundamental concerns about patient welfare may persist, preventing professionals from perceiving it as useful. Second, resistance to change in geriatric institutions may be rooted in established care philosophies that emphasize human touch and presence as therapeutic tools, particularly for dementia and end-of-life care (Zulfiqar et al., 2018). This suggests that in geriatric contexts, resistance operates at a deeper ideological level that PEOU cannot address. Third, the interdisciplinary nature of geriatric care teams may amplify resistance, as concerns from one professional group can influence the entire team’s perceptions (Almathami et al., 2020). Additionally, the multidimensional nature of resistance to change, comprising routine seeking, emotional reaction, short-term focus, and cognitive rigidity (Oreg et al., 2008) may contribute to this complexity, with each dimension potentially requiring different intervention strategies.

This finding is inconsistent with some recent studies that have investigated the role of PEOU as a mediator in the context of technology adoption in healthcare. For instance, other research found that PEOU mediated the relationship between technology anxiety and PU in their integrative review of predictive factors and intervention programs for technology acceptance in healthcare (Gücin & Berk, 2015). However, their studies primarily focused on acute care settings where efficiency benefits are more immediately apparent, unlike geriatric care where relationship-based care dominates. In contrast, the present study’s findings align with other study, who found that PEOU did not mediate the relationship between resistance to change and PU in their systematic review of factors impacting clinicians’ adoption of mobile health tools (Jacob et al., 2020). It identified this pattern specifically in “high-touch” specialties managing vulnerable populations, supporting our geriatric-specific findings (Jacob et al., 2020).

### 4.1. Limitations and Future Research Directions

Several methodological limitations should be considered when interpreting these findings. Most importantly, this study examines only healthcare professionals of telemedicine adoption without the patients’ perspectives in geriatric care. The cross-sectional design prevents establishing causal relationships between behavioral factors and technology adoption intentions, limiting our understanding of how these relationships develop over time. The convenience sampling approach, while achieving good response rates, may not fully represent all healthcare professionals in Israeli GMCs. Additionally, self-report measures of behavioral intentions may not accurately predict actual technology use due to the well-documented intention-behavior gap. The study’s focus on four Israeli GMCs limits generalizability to other healthcare systems with different organizational structures, funding models, and cultural contexts.

Furthermore, this study did not examine several potentially important factors that may influence telemedicine adoption. These include demographic variables such as age and years of experience, individual personality traits, technological prejudice, and the impact of previous negative experiences with technology implementations (Bhattacherjee & Hikmet, 2007; Xue et al., 2015). Future research should investigate how these additional factors interact with the behavioral variables examined in this study to provide a more comprehensive understanding of telemedicine adoption in geriatric care settings.

Future research should include: patient-provider dyadic studies examining both perspectives on adoption; longitudinal tracking from initial exposure through sustained use to reveal behavioral evolution; objective usage data to validate self-reported intentions; multi-level analyses of organizational and team influences; international comparisons identifying universal versus culture-specific factors; and experimental interventions testing strategies for building self-efficacy, managing social influence, and addressing technology anxiety to provide evidence-based implementation guidance.

### 4.2. Theoretical and Practical Implications

Theoretically, our findings extend TAM by demonstrating that negative inhibitors (anxiety, resistance) operate differently than positive facilitators (self-efficacy, subjective norms) in specialized healthcare contexts. This suggests TAM requires modification for context-specific factors like geriatric care’s emphasis on human touch that override technical usability.

Practical implications include: First, training programs should build self-efficacy through hands-on practice and graduated skill-building. Second, organizations should leverage subjective norms by supporting departmental technology champions who model successful use and provide peer support. Third, given PU’s strong mediating role, organizations must clearly demonstrate telemedicine’s concrete benefits for patient outcomes and practice efficiency.

## 5. Conclusions

This study reveals crucial behavioral mechanisms underlying telemedicine adoption in geriatric care settings. The differential pathways of positive and negative factors—with self-efficacy and subjective norms operating through PEOU as confirmed by our mediation analyses while anxiety and resistance do not as evidenced by non-significant indirect effects—indicate that behavioral interventions must be tailored to specific psychological states. The model’s strong explanatory power (R^2^ = 0.65) for intention to use telemedicine confirms that behavioral factors are key determinants of technology adoption intentions in geriatric medical centers.

These results extend previous TAM applications (Davis, 1989; Venkatesh et al., 2003) by demonstrating context-specific variations in geriatric care settings. Healthcare organizations should implement evidence-based behavioral strategies that build confidence through mastery experiences consistent with Bandura’s self-efficacy theory, stimulate social influence through peer modeling, and address anxiety through targeted psychological support rather than merely improving technology interfaces given our finding that PEOU does not mediate the anxiety–PU relationship.

## Figures and Tables

**Table 1:** Background characteristics.

Characteristic	Categories	Frequency	%
Gender	Women	253	62.3
	Men	127	31.3
Marital Status	Single	72	17.5
	Married/In a relationship	264	65.0
	Divorced	40	9.9
	Widowed	5	1.2
Level of Religiosity	Secular	150	36.9
	Traditional	133	32.8
	Religious	74	18.2
	Very Religious	9	2.2
Religion	Jewish	186	45.8
	Muslim	156	38.4
	Christian	19	4.7
	Druze	2	0.5
	Other/non	3	0.7
Children	Yes	272	67.0
	No	104	25.6
Number of Children (if any)	1–2	149	36.7
	3–4	99	24.4
	4 and above	11	2.9
Job Description	Physician	42	10.3
	Nurse	225	55.4
	Social Worker	13	3.2
	Physiotherapist	32	7.9
	Speech Therapist	11	2.7
	Occupational Therapist	15	3.7
	Clinical Dietitian	12	3.0
Work in Shifts	Morning	139	34.2
	Evening/Night Shifts	222	54.7

Note: The total number of participants for each characteristic does not always add up to 406 for missing values.

**Table 2:** Fornell–Larcker Criterion, Correlations and Square Root of AVE.

Variables	1	2	3	4	5	6	7
1. Self-efficacy for using telemedicine	**0.857**						
2. Subjective norms towards telemedicine	0.486	**0.863**					
3. Anxiety about using telemedicine	−0.328	−0.074	**0.831**				
4. Resistance to technological changes at work	−0.320	−0.007	0.627	**0.797**			
5. Perceived usefulness	0.495	0.618	−0.343	−0.302	**0.903**		
6. Perceived ease of use	0.633	0.569	−0.367	−0.328	0.722	**0.867**	
7. Intention to use telemedicine	0.482	0.518	−0.307	−0.272	0.762	0.734	**0.947**

**Table 3:** Direct and Indirect Effects of Model Variables.

	B	SE	LLCI, ULCI
Perceived usefulness -> intention to use telemedicine	0.48	0.06	0.35, 0.61
Perceived ease of use -> intention to use telemedicine	0.46	0.08	0.30, 0.63
Perceived ease of use -> perceived usefulness	0.61	0.08	0.44, 0.78
Self-efficacy for using telemedicine -> perceived usefulness	−0.04	0.05	−0.14, 0.05
Subjective norms towards telemedicine -> perceived usefulness	0.37	0.06	0.24, 0.51
Anxiety about using telemedicine -> perceived usefulness	−0.08	0.04	−0.17, 0.01
Resistance to technological changes at work -> perceived usefulness	−0.11	0.07	−0.26, 0.02
Self-efficacy for using telemedicine -> perceived ease of use	0.29	0.05	0.18, 0.39
Subjective norms towards telemedicine -> perceived ease of use	0.34	0.05	0.24, 0.45
Anxiety about using telemedicine -> perceived ease of use	−0.10	0.05	−0.212, 0.00
Resistance to technological changes at work -> perceived ease of use	−0.12	0.07	−0.26, 0.01
Self-efficacy for using telemedicine -> perceived ease of use -> perceived usefulness	0.17	0.04	0.09, 0.25
Subjective norms towards telemedicine -> perceived ease of use -> perceived usefulness	0.21	0.03	0.14, 0.28
Anxiety about using telemedicine -> perceived ease of use -> perceived usefulness	−0.06	0.03	−0.13, 0.00
Resistance to technological changes at work -> perceived ease of use -> perceived usefulness	−0.07	0.04	−0.163, 0.01
perceived ease of use -> perceived usefulness -> intention to use telemedicine	0.29	0.05	0.19, 0.39

## Data Availability

The original contributions presented in this study are included in the article. Further inquiries can be directed to the corresponding author.
